# Resveratrol Modulates Diabetes-Induced Neuropathic Pain, Apoptosis, and Oxidative Neurotoxicity in Mice Through TRPV4 Channel Inhibition

**DOI:** 10.1007/s12035-024-04311-4

**Published:** 2024-07-08

**Authors:** Haci Ömer Osmanlıoğlu, Mustafa Nazıroğlu

**Affiliations:** 1 Department of Anesthesiology and Reanimation, Medical Faculty, Suleyman Demirel University, 32260 Isparta, Türkiye; 2 Neuroscience Application and Research Center (NOROBAM), Suleyman Demirel University, Isparta, Türkiye; 3 BSN Health, Analyses, Innovation, Consultancy, Organization, Agriculture, and Industry Ltd, Isparta, Türkiye; 4 Department of Biophysics, Medical Faculty, Suleyman Demirel University, Isparta, Türkiye

**Keywords:** Apoptosis, Diabetic peripheral neuropathy, Oxidative injury, Resveratrol, TRPV4 channel

## Abstract

Diabetic peripheral neuropathy (DPN) is caused by several factors, including reactive free oxygen radicals (ROS)-induced excessive Ca^2+^ influx. Transient receptor potential (TRP) vanilloid 4 (TRPV4) is a member of the Ca^2+^-permeable TRP superfamily. Resveratrol (RESV) has been extensively utilized in TRP channel regulation due to its pharmacological properties, which include antioxidant and TRP inhibitory effects. The protective function of RESV and the contribution of TRPV4 to streptozotocin (STZ)-induced neuropathic pain in mice are still unclear. Here, we evaluated the effects of RESV through the modulation of TRPV4 on Ca^2+^ influx, ROS-mediated pain, apoptosis, and oxidative damage in the mouse dorsal root ganglion (DRGs). From the 32 mice, four groups were induced: control, RESV, STZ, and STZ + RESV. We found that the injection of RESV reduced the changes caused by the STZ-induced stimulation of TRPV4, which in turn increased mechanical/thermal neuropathic pain, cytosolic Ca^2+^ influx, TRPV4 current density, oxidants (lipid peroxidation, mitochondrial ROS, and cytosolic ROS), and apoptotic markers (caspase-3, -8, and -9). The RESV injection also increased the STZ-mediated reduction of viability of DRG and the amounts of glutathione, glutathione peroxidase, vitamin A, β-carotene, and vitamin E in the brain, erythrocytes, plasma, liver, and kidney. All of these findings suggest that TRPV4 stimulation generates oxidative neurotoxicity, neuropathic pain, and apoptosis in the STZ-induced diabetic mice. On the other hand, neurotoxicity and apoptosis were reduced due to the downregulation of TRPV4 carried out through the RESV injection.

**Graphical Abstract:**

An overview of how resveratrol (RESV) inhibits TRPV4 in mice to modulate the effects of diabetes mellitus-induced diabetic peripheral neuropathy (DPN). Ruthenium red (RuR) inhibits TRPV4, while GSK1016790A (GSK) and reactive free oxygen radicals (ROS) activate it. In the mitochondria of DRGs, the glucose oxidation brought on by diabetes mellitus (STZ) causes an intracellular free Ca^2+^ and Zn^2+^ influx excess that is dependent on TRPV4. The administration of STZ leads to the DRG becoming more depolarized (ΔΨm), which in turn causes an increase in mitochondrial ROS, apoptosis, and caspases (caspase-3, caspase-8, and caspase-9) by downregulating enzymatic (glutathione peroxidase, GSH-Px) and non-enzymatic (glutathione (GSH), vitamin A, and vitamin E) antioxidants. The mice’s molecular pathways were diminished by the RESV injections. (Increase (↑); diminish (↓))

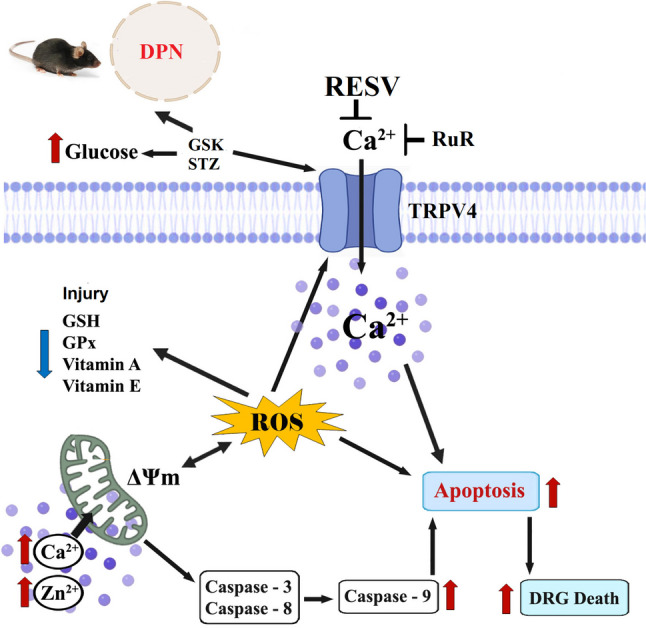

## Introduction

Diabetic peripheral neuropathy (DPN) causes 2.4 to 78.8% worldwide in people with diabetes mellitus [1]. DPN is a serious clinical problem that lowers the standard of living and for which there is currently no safe and efficient medication solution [2]. DPN is typified by generalized destruction to tiny nerve fibers, notably those of the Aδ and C type of the spinal cord dorsal column, and is characterized by burning, electric, stabbing, or tingling neuropathic pain that begins in the lower limbs. Dorsal root ganglions (DRGs) were found to be a safe and effective neuromodulation modality that improves painful symptoms in DPN [2, 3]. DRGs enhanced oxidative neurotoxicity as a result of diabetes-related metabolic and vascular problems [4, 5]. The cause of DPN has been linked to excessive amounts of intracellular free oxygen radicals (iROS) and mitochondria-derived free oxygen radicals (mtSOX) and a decrease of non-enzymatic and enzymatic ROS scavengers [4–7]. An upregulation in intracellular free Ca^2+^ amount ([Ca^2+^]_i_) is an essential contributory component to the mtSOX amplification in hyperglycemia [8, 9]. The discovery of novel analgesic adjuvants is essential to the effective attenuation of DPN. Treatment for chronic pain may include pharmacological targeting of transient receptor potential (TRP) channels [10, 11].

It has been demonstrated that the TRP vanilloid (TRPV4) channel, which is a member of the TRP super group, is a widely expressed, polymodally gated, non-selective cation channel that is necessary for several biological processes, including DPN and insulin synthesis [10, 11], although conflicting reports are also present on Ca^2+^ signaling and insulin secretion [12, 13]. In oxidative pain conditions, TRPV4 was discovered to be implicated in mechanical hyperalgesia and hypotonicity-induced nociception [10, 14]. Several synthetic pharmacological compounds, such as GSK1016790A (GSK) and oxidative stress, stimulate TRPV4 [14–16]. Following DRG diabetic oxidative damage, there was a considerable increase in the number of TRPV4-immunoreactive sensory neurons in addition to the co-localization of TRPV4 in small/medium diameter sensory neurons of the DRGs [10]. In mouse white adipocytes, TRPV4 also functions as a negative regulator of oxidative metabolism [17], and it is activated in DRGs by mtSOX and nitric oxide-mediated pathways [10, 18]. Chemotherapy-induced peripheral neuropathy and mtSOX production were induced by the stimulation of TRPV4 [19]. Previous studies used non-selective TRPV4 inhibitors, including ruthenium red (RuR) [20, 21]. TRPV4 has been shown to be significantly expressed in DRGs [11, 22], and the DPN modulator action of GSK through the inhibition of TRPV4 was reported in the DRGs of rodents [23]. Natural plants and fruits, including grapes, *Veratrum*, and peanuts, contain the antioxidant polyphenol resveratrol (RESV). In the treatment of numerous neurological conditions, RESV has a number of beneficial advantages, including decreasing inflammation, regulating blood sugar, minimizing oxidative stress injury, and enhancing insulin resistance [24]. Improving mitochondrial activity, scavenging free radicals such as lipid peroxidation (LPx), iROS, and mtSOX, and decreasing tissue oxidative damage are all accomplished by RESV. Additionally, it inhibits TRP channels that are susceptible to ROS, such as TRP vanilloid 1 (TRPV1), TRP canonical 3 (TRPC3), and TRP melastatin 2 (TRPM2) [25–27]. Additionally, recent studies [28–30] detailed the modulator effects of RESV on DPN in cell lines and experimental animals. In human pulmonary artery endothelial cells [31] and SH-SY5Y neuronal cells [21], TRPV4 stimulation led to an increase in mtSOX and apoptosis; however, the increases were mitigated by the administration of antioxidants like carvacrol [21]. Hence, the severity of DPN was modulated by the inhibition of oxidative stress-dependent TRP channel inhibitions. Nevertheless, there is no data in the literature regarding the anti-DPN and antidiabetic effects of RESV through TRPV4 suppression.

It is commonly recognized that in cells and experimental animals, an increased Ca^2+^ influx caused by diabetes mellitus results in oxidative stress and DPN. Although their molecular processes are still unclear, the protective effects of RESV against DPN in rats and cells have been studied [28–30]. To our knowledge, little study has been done on the effects of RESV on TRPV4-mediated DPN, oxidative stress, and apoptosis in diabetic (streptozotocin, STZ) model mice. The current study had two objectives. Initially, we investigated whether TRPV4 activation in the DRG of mice with diabetes mellitus produced pain-like responses, oxidative neurotoxicity, and apoptosis. Secondly, since RESV injection is known to inhibit oxidative neurotoxicity and apoptosis by modifying ROS-sensitive TRP channels in the DRGs of laboratory animals [25–27], we examined the protective effects of RESV through the attenuation of TRPV4 on the oxidative injury and programmed cell death (apoptosis) caused by diabetes mellitus. Using DPN measurement, oxidant, and apoptotic techniques, we were able to show that diabetes mellitus produces mechanical and thermal DPN by means of TRPV4 stimulation. Moreover, we found that whereas oxidative damage and apoptosis generated in DRGs target TRPV4 to produce DPN, RESV reduced DPN, oxidative neuro-injury, and apoptosis via the modulation of TRPV4 in the DRG of mice with diabetes mellitus.

## Materials and Methods

### Mice

A total of 16 male and 16 female C57BL/6j mice, weighing between 20 and 25 g and 8 weeks old, were acquired from the Research Center of the Experimental Animal University of Burdur Mehmet Akif (MAKU), Türkiye. The mice were kept in a room with climate control that included light (12:12 h) and heat (21 ± 1 °C). For a total of 26 days, they were given free tap water and ad libitum commercial feed mixture of Kortkutelim Animal and Human Food Inc. (Korkuteli, Antalya, Türkiye). According to the guidelines established by MAKU, each experiment was assessed and approved by the Local Animal Care Committee of MAKU (date, 17.11.2022; meeting number, 108; decision number, 993). There were only a limited number of mice used in this research.

### Experimental Groups

**Fig. 1:**
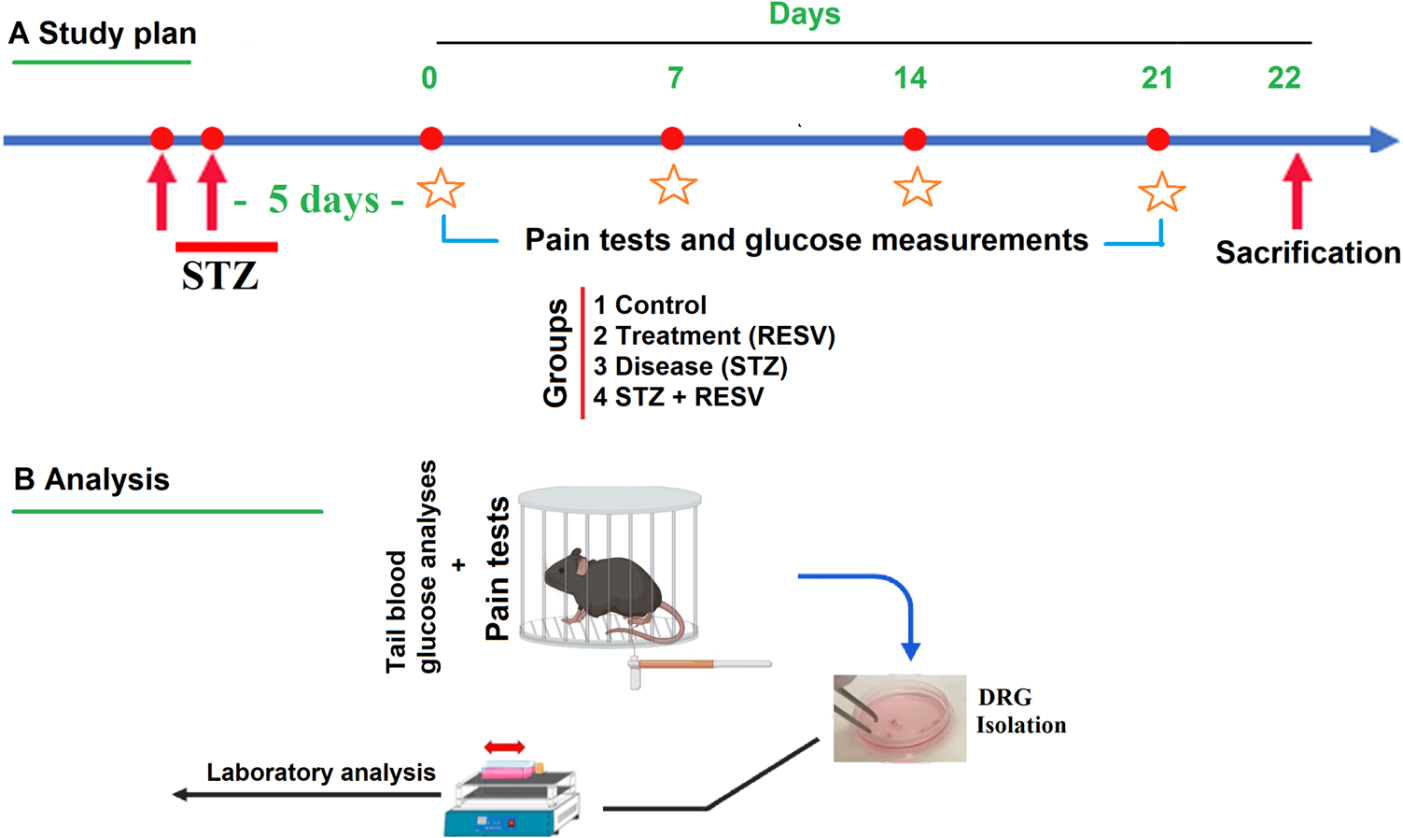
Study plan. Four groups of 32 mice each were generated: control, STZ + RESV, RESV, and STZ. Following the injection of a single dose of freshly produced STZ (45 mg/kg) in citrate buffer to induce diabetes, the RESV and STZ + RESV groups received intraperitoneal injections of RESV (25 mg/kg) for 21 days. On days 0, 7, 14, and 21 of the study, tail blood glucose levels and pain intensity were evaluated. DRG samples were collected for analysis on the 22^nd^ day

A total of 32 mice (*n* = 8) were used in the four groups as control (CN), RESV, STZ, and STZ + RESV. Each group contained 4 female and 4 male mice. The CN group received intraperitoneal physiological saline for 3 weeks. The RESV group received intraperitoneal RESV (25 mg/kg) for 3 weeks [30]. In STZ groups, fresh STZ (Cat # S0130, Sigma-Aldrich, St. Louis, MO, USA) in a fresh citrate buffer (0.1 mmol/l and pH 4.5) at a concentration of 45 mg/kg was injected intraperitoneally into the mouse in the STZ in order to induce diabetes mellitus and hyperalgesia [5, 8, 9]. To prevent diabetes mellitus and hyperalgesia, mice in the STZ + RESV groups were given intraperitoneal RESV (25 mg/kg) for 3 weeks following a single intraperitoneal STZ injection [5, 8, 9] (Fig. 1A and B). As a diabetic approach, mice with tail vein levels of blood glucose ≥ 250 mg/dl, or hyperglycemia, were identified. A standard RESV solution was prepared in DMSO. To get the appropriate concentrations, they were diluted in 0.5 ml of physiological saline solution. The stock solutions of TRPV4 agonist (GSK) (Cat # G0798-10 mg, Sigma-Aldrich) and antagonist ruthenium red (RuR) (Cat # 557,450) in several tests were also prepared in DMSO. Following the DRG isolation of mice, an incubation with GSK (100 nM) activated the TRPV4 channel, while an incubation with RuR (1 µM) blocked it.

### The Preparation of DRG and Brain Cortex Samples

The laboratory animals were sacrificed following their anesthesia with ketamine and xylazine. In the lumbar region of the mouse, the DRGs located in the L4 and L6 regions were dissected. Fluorescence confocal microscope with laser scanning (CLSFM, LSM-800, Zeiss, Oberkochen, Germany) images and spectrophotometer antioxidant analyses were performed on the small/medium diameter sensory neurons of whole DRGs (Fig. 1B). For conventional patch-clamp and plate reader investigations, the connected nerves and surrounding connective tissues of DRGs were cut away. Then, as previously reported [5, 6, 8], they were subjected to additional processes such as trypsin type III enzyme and type XI collagenase incubations. After being treated with 5 mg laminin per milliliter (Cat # 23,017,015, ThermoFisher Scientific, Waltham, MA USA) and poly-DL-ornithine (Cat # P4957, 0.1 mg/ml; Sigma-Aldrich), dissociated cells (2 × 10^3^ neurons/ml) were seeded on coverslips in the DMEM (Cat # D5671-500 ml, Sigma-Aldrich) medium mixture and maintained at 37 °C in the cell culture conditions (95% air/5% CO_2_).

In addition to the DRGs, erythrocytes, plasma, liver, kidney, and cerebral cortex were obtained from the mice. The erythrocytes, cerebral cortex, liver, kidney, and plasma samples were examined in each group to ascertain the antioxidant and LPx concentrations because there were insufficient DRG sample amounts for the LPx and antioxidant analyses.

### Assessment of the Hind Paw Withdrawal Threshold and Glucose

To assess the sensitivity of mice to pain, von Frey monofilaments (20PC Aesthe, Muromachi Kikai Ltd. Tokyo, Japan) and a hot plate were utilized. As previously mentioned, [5, 6, 8] we measured the mouse’s paw withdrawal threshold (von Frey) and thermal paw withdrawal delay (hot plate) before and after STZ and RESV were administered. The hind paw withdrawal threshold tests (using a Von Frey and a hot plate) and the tail blood glucose analyses (eBsensor, Visgener Inc., Hsinchu City, Taiwan) were conducted on the baseline, seventh, fourteen, and twenty-first days in each of the four groups. All behavioral tests were conducted in the absence of observers.

### Conventional Whole-Cell Patch-Clamp Recordings

**Fig. 2:**
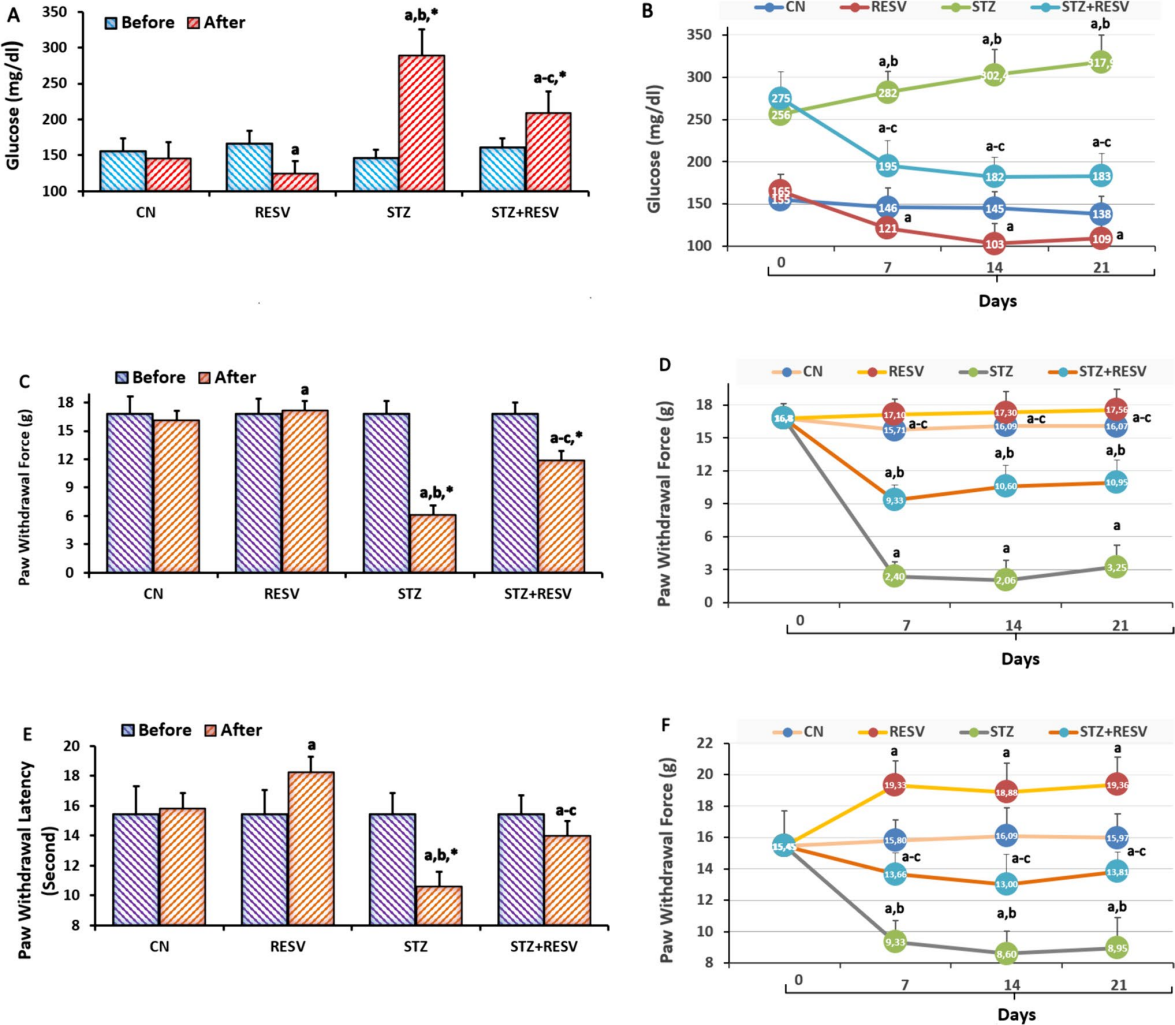
In diabetic mice, the 3-week injection of 25 mg/kg of RESV reduced the rise in pain intensity and blood glucose levels caused by STZ (mean ± SD; *N* = 8). **A** and **B** The glucometer was used to measure the blood glucose levels in the tail. The von Frey monofilament (**C** and **D**) and hot plate (**E** and **F**) were used to measure each mouse’s pain levels. (^a^*p* < 0.05 vs CN; ^b^*p* < 0.05 vs CN + GSK; ^c^*p* < 0.05 vs STZ + GSK. *p < 0.05 vs ‘Before’ groups)

A puller (PC-10, Narishige Group, Tokyo, Japan) was used to construct patch pipettes (GB150F-10, Science Products GmbH, Hofheimer, Germany), and conventional whole-cell patch-clamp methods for recording were applied to determine the TRPV4 currents in the small- and medium-sized DRGs (Fig. 2G and H) [6, 8]. The access of whole-cell recording electrode cell resistances ranged from 2 to 6 MΩ. The following chemicals (in mM) were used to make the extracellular (patch chamber) solution: CaCl_2_ (1), D-glucose (10), HEPES (10), KCl (5), NaCl (145), and MgCl_2_ (1) (pH, 7.4). Instead of using Na^+^, we prepared the Na^+^-free extracellular solution by adding 150 mM *N*-methyl-d-glucamine (NMDG^+^) and adjusting the pH with HCl. The intracellular (pipette) solution was prepared with the following chemicals (in mM): cesium glutamate (145), EGTA (8), HEPES (10), MgCl_2_ (1), and NaCl (8); the pH was adjusted to 7.2 using CsOH. The osmolarities of solutions were maintained between 300 and 320 mOsmol/l by using an Osmomat 030 osmometer (Gonotec, Berlin, Germany). The intracellular physiologic Ca^2+^ concentrations were produced using the MAXC program (http://www.stanford.edu/~cpatton/maxc.html). Every experiment was conducted at 22 ± 4 °C, room temperature. We waited until the currents returned to their basal (control) values after triggering the entire cell arrangement. We employed NMDG^+^ to measure the live or dead cell currents. We carried out the GSK applications when we noticed an inward current in the neurons following the application of RuR or NMDG^+^. At − 60 mV, the voltage was clamped. The EPC10 patch-clamp amplifier (Multi Channel Systems MCS GmbH HEKA, Stuttgart, Germany) was used to measure the currents. RuR (1 µM) suppressed TRPV4 in the DRGs, while GSK (100 nM) activated it. The current intensity (pA/pF) value was utilized to indicate the whole-cell patch-clamp data.

### Detection of [Ca^2+^]_i_ Alterations in the DRGs

In order to quantify variations in [Ca^2+^]_i_ fluorescence intensity, the green images of whole DRGs in the bottom glass plates (Cat # P35G-1.5–14-C, MatTek Company, Ashland, MA, USA) were captured using the CLSFM connected to the Axio Observer 7 inverted microscope. Before being stimulated by an argon laser at 488 nm, the DRGs were stained for an hour using 1 µM Fluo-3/AM dye (Cat # ab145254, Abcam Inc., Cambridge, UK) [6]. Using a special program (ZEN, blue edition), differences in the intensity of fluorescence in previously stored images were examined. The results of the [Ca^2+^]_i_ fluorescence intensity were displayed in arbitrary units (a.u.).

### The Investigations of iROS and Mitochondrial Function in the DRGs

In DRGs labeled with JC-1 (Cat # T3168, Thermo Fisher), mitochondrial depolarization of the membrane (mtMD) was observed as a decrease in the potential of the mitochondrial membrane using the CLSFM connected to an objective (Plan-Apochromat 20 × /0.8). After being cleaned and then treated with 2 µM JC-1 for 15 min at incubator temperature (37 °C), the DRGs from the different injection groups were placed on confocal microscopy for visualization in the 35-mm dishes. The JC-1 was stimulated with a diode argon laser operating at 488 nm. A rise in mtMD, which was associated with a decrease in the JC-1 ratio, and an increase in orange color demonstrated a decrease in mitochondrial membrane potential [32].

MitoSOX Red reagent (Cat # M36008 and ThermoFisher), a mitochondrial superoxide indicator for live-cell imaging, was used to determine the generation of ROS in the mitochondria in the 35-mm dishes. MitoSOX (5 mM) was added to treated cells and incubated for 20 min at 37 °C in the dark. The diode argon laser was used to stimulate the mtSOX Red at 561 nm. CLSFM was used to obtain the images.

A non-fluorescent dye (DCFH-DA) (Cat # ab113851, Abcam, Istanbul, Türkiye) can easily pass across the membranes of cells. Once inside the cell, the probe DCFH-DA was employed to determine the amount of iROS generated. This probe generates dichlorofluorescein (DCF) when it is oxidized, and its fluorescence is directly correlated with the level of ROS generated [33]. A diode argon laser emitting at 488 nm was used to stimulate the DCFH-DA of whole DRGs in the 35-mm dishes. The green DCF pictures were then recorded in the CLSFM.

The obtained red (mtSOX), orange (JC-1), and green (DCFH-DA) changes of the images in fluorescence intensity were measured as a.u. using the ZEN program. Previous investigations [25, 34] provided data on mtSOX, mtMD, and iROS.

### Assessments of Caspase Levels, Apoptosis, and Cell Viability

Six-well plates containing 100 µl of MTT (5 mg/ml) in PBS were used to seed DRGs at a density of 2 × 10^3^ neurons per milliliter. The plates were then incubated for 4 h at 37 °C. To try to dissolve the formazan crystals, 500 µl of dimethyl sulfoxide (DMSO; Sigma-Aldrich) was added. After shaking the six-well plate for a minute, its absorbance was detected at 492 nm using a microplate reader (Infinite 200 PRO, Tecan Inc., Männedorf, Switzerland). The commercially available APOPercentage apoptosis evaluation kit from Biocolor Ltd. (Cat # A1000, County Antrim, UK) was utilized to measure the amount of DRG apoptosis using an Infinite 200 PRO microplate reader [25, 34].

Active caspases -3, -8, and -9 predominantly cleaved fluorogenic substrates (Ac-DEVD-AMC, Ac-IETD-AFC, and Ac-LEHD-AFC) (Bachem AG., Bubendorf, Switzerland) to the AMC and AFCs following incubation with apoptotic cell lysates in black plates, respectively. The cleavage levels of free AMC and AFCs were determined using the microplate reader (Infinite 200 PRO) at the excitation (360–400 nm) and emission (460–505 nm) wavelengths [25, 34].

The total protein amount or optical density/fluorescence intensity values measured in the DRGs were used to calculate the 100% expression of the DRG viability, apoptosis, and caspases -3, -8, and -9.

### Propidium Iodide (PI)-Positive Cell Percentage Detection

PI (Cat # P1304MP, ThermoFisher Scientific) and Hoechst 33342 (Cat # H3570, ThermoFisher Scientific) were used to calculate the cell death percentage. The neurons in the medium of dishes were stained with Hoechst 33342 (8.1 mM) and PI (1.5 mM). After incubation for 20 min, blue (Hoechst), red (PI), 2.5D, and bright field (BF) pictures were taken in the CCD Axiocam 702 camera and Axio Observer.Z1/7 inverted microscope (Zeiss). The percentage of PI-positive cells in the CCD camera evaluation was established by manually counting every single cell in the collected cells.

### Analysis of Intracellular Glutathione (iGSH) and Free Zn^2+^ ([Zn^2+^]_i_)

The iGSH measurement assay ThiolTracker Violet (Cat # T10095, ThermoFisher Scientific) was utilized to evaluate the GSH concentrations in whole DRGs. The green pictures of ThiolTracker Violet in DRGs were recorded using the ZEN software (blue edition 3.2) and the CLSFM microscope (Ext, 405 nm; Emi, 526 nm) [6, 16].

The concentrations of [Zn^2+^]_i_ in the DRGs were stained utilizing RhodZin3/AM (1 µM per ml) (Cat # T24195, ThermoFisher Scientific), a fluorescent probe for Zn^2+^ labeling. Using the CLSFM microscope (Emi, 526 nm; Ext, 404 nm) and the ZEN software, green pictures of the RhodZin3/AM cells were captured.

The intensity of fluorescence changes in the green-colored images was determined utilizing the ZEN program (blue edition 3.2), and findings were shown as a.u.

### The Measurements of Lipid Peroxidation and Antioxidants in Brain Samples

The ultrasonic homogenizer (HD2200, Bandelin, Berlin, Germany) was utilized for preparing the brain cortex, liver, and kidney homogenates [6, 35]. Disodium EDTA, an anticoagulant, was used to extract plasma samples from the whole blood tube using centrifugation (500 g for 5 min). Hemolysate of erythrocytes was made by washing the residual erythrocytes at the bottom of the tube with physiological saline [6, 35]. Using a spectrophotometer (UV-1800, Shimadzu, Kyoto, Japan), the optic density variations of total protein, LPx (at 532 nm) [36], reduced glutathione (rGSH) (at 412 nm) [37], and glutathione peroxidase (GSH-Px) (at 412 nm) [38] in the brain cortex were evaluated by hand. A detailed discussion of the analyses was given in previous studies [6, 35]. While the amounts of LPx and rGSH are expressed in µmol/gram protein, the GSH-Px activity in the brain homogenates was reported as IU/gram protein.

In accordance with previous studies [6, 39], the amounts of vitamins A, β-carotene [40], and E [39] in the cerebral cortex were measured by manually utilizing the UV-1800 spectrophotometer set at 325 nm (retinol), 450 nm (β-carotene), and 532 nm (α-tocopherol). To determine vitamins A and E, the standard solutions—all-trans-retinol, β-carotene, and α-tocopherol—were dissolved in hexane. The vitamin concentrations in wet tissue (µmol/g) were reported for the liver, brain, and kidney. Plasma vitamin concentrations were indicated as µmol/l.

### Statistics

After calculating the mean ± standard deviation (SD) values from the mean data, Fisher’s least significant difference (LSD) test and the SPSS program (Version 22.0, Chicago, IL, USA) were used to determine whether there was any statistical significance in any of the groups. One-way analysis of variance (ANOVA) was used to determine whether there was statistical significance (*p* < 0.05) in the groups.

## Results

### Blood Glucose Levels, Mechanical Hyperalgesia, and Thermal Hyperalgesia Increases Caused by STZ Were Reduced by the Injections of RESV

The evaluations of mechanical hind paw withdrawal force (von Frey), heat withdrawal latency (hot plate), and glucometer, respectively, showed a progressive increase for 21 days following the injection of STZ in the blood glucose (Fig. 2A and B), von Frey (Fig. 2C and D), and hot plate (Fig. 2E and F). Therefore, when compared to the STZ alone, the treatments with RESV resulted in a decrease in mechanical hind paw withdrawal force, blood glucose levels, and heat hind paw withdrawal latency in the RESV and STZ + RESV groups (*p* < 0.05). On pain intensity and glucose levels, we did not see the effects of gender differences in the mice (*p* > 0.05).

### RESV Attenuated the Increase in TRPV4 Current Densities in the DRGs Caused by STZ

**Fig. 3:**
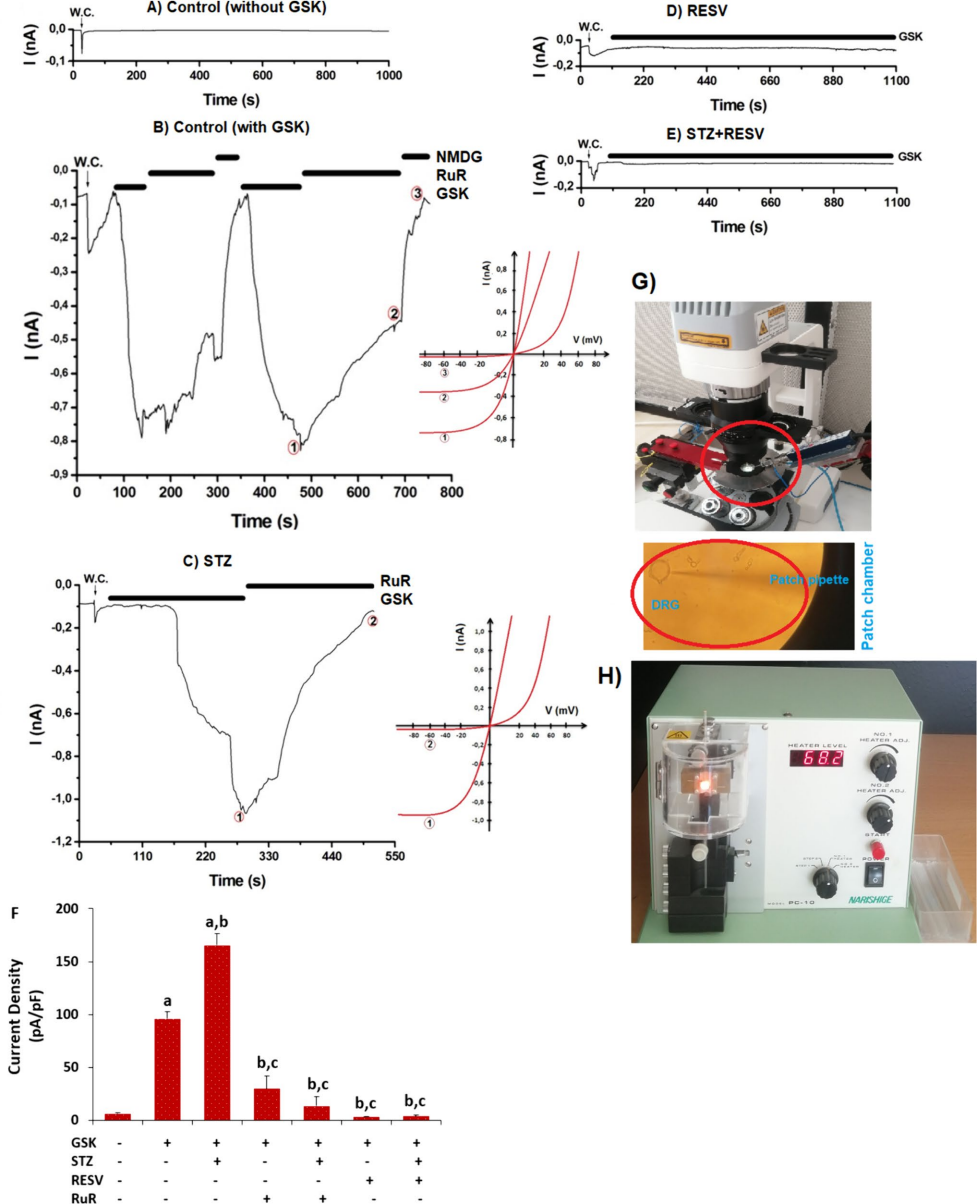
The 3-week RESV injections (25 mg/kg) attenuated the STZ-mediated elevation of TRPV4 current density in the DRGs (mean ± SD; *N* = 8). In the DRGs, TRPV4 was stimulated by GSK (100 nM), whereas RuR (1 µM) inhibited it. **A** Control (CN) (without GSK agonist). **B** The RuR/NMDG group with CN + GSK. **C**. STZ group containing RuR and GSK. The current (I)–voltage (V) correlation of **B** and **C**, respectively, is shown. **D** RESV but in the absence of STZ. **E** RESV in combination with STZ. **F** The TRPV4 current mean densities. **G** Patch-clamp setup for a whole cell (W.C.). **H** Puller apparatus. (^a^*p* < 0.05 vs CN; ^b^*p* < 0.05 vs RESV; ^c^*p* < 0.05 vs STZ + RESV)

TRPV4 current densities were indicated as pA/pF in the present study. In the DRGs of CN without GSK stimulation, there was limited current (Fig. 3A). Nevertheless, the addition of extracellular GSK (100 nM) activated the TRPV4 of DRGs in the patch chamber (Fig. 3B). As expressed in pA/pF, the mean TRPV4 current densities in the CN + GSK group (96.05) were higher than those in the CN (6.41) (Fig. 3A and F) (*p* < 0.05). The STZ + GSK group had higher mean densities of TRPV4 currents (165.33) (Fig. 3C) than the CN + GSK group (*p* < 0.05). In comparison to the CN + GSK (96.05) and STZ + GSK (165.33) groups, the TRPV4 current densities were lower in the CN + GSK + RuR (29.74) and STZ + GSK + RuR (13.72) groups (*p* < 0.05). By the treatments of RESV with STZ (3.71) without (Fig. 3D)/with (Fig. 3E) STZ, we observed limited currents as control as in the DRGs. In the control group, the TRPV4 currents were observed in 0.99 ± 0.41 min, while in the STZ groups, they were obtained in 0.57 ± 0.13 min. Thus, STZ was used to stimulate TRPV4 more quickly. The increased current density in the DRGs carried on by STZ is attributed to the TRPV4, according to the present electrophysiological data.

### The Amount of [Ca^2+^]_i_) Increased Due to STZ-Induced TRPV4 Activation but Was Reduced in the DRGs Following RESV Injection

**Fig. 4:**
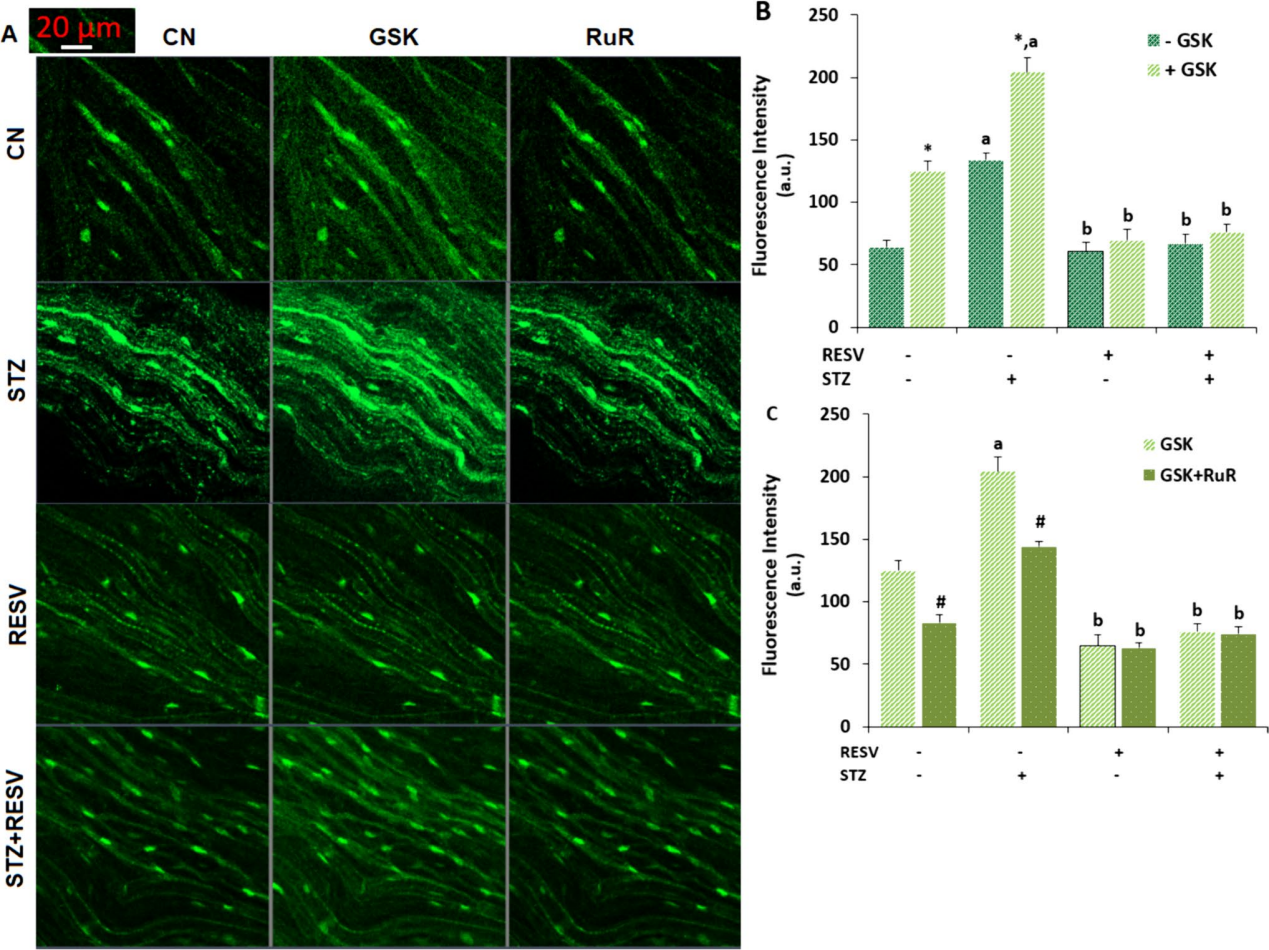
The injection of RESV (25 mg/kg for three weeks) reduced the increase in [Ca^2+^]_i_ levels caused by STZ blocking the TRPV4 channel in the mouse DRGs (*N* = 8 looking at 9–13 DRGs and mean ± SD each). **A** Following a 60- to 90-min labeling period of the whole DRG neurons with Fluo 3/AM (1 µM), the green images from each of the four groups were taken using a CSLFM that had a 20 × 0.8 objective. The [Ca^2+^]_i_ fluorescence intensity changes in each of the four groups after receiving GSK and RuR therapy, respectively, are displayed in** B** and **C**. Arbitrary unit (a.u.), The scale bar is 20 µm. [(*p < 0.05 vs CN; ^a^*p* < 0.05 vs without GSK stimulation group (− GSK); ^b^*p* < 0.05 vs STZ; ^**#**^*p* < 0.05 vs GSK stimulation group (+ GSK)]

The amount of [Ca^2+^]_i_ in the DRGs was greater in the STZ group than in the CN and RESV groups (Fig. 4A and B). However, treatment with a TRPV4 antagonist (1 µM RuR) effectively suppressed the amount in neurons (*p* < 0.05) (Fig. 4A and C). According to the Fluo-3/AM data, the [Ca^2+^]_i_ amount in DRGs of the RESV group was not increasing in the absence of the STZ (*p* ≥ 0.05). Nonetheless, the RESV injections considerably lowered the rise in [Ca^2+^]_i_ concentration in the neurons of STZ + RESV (*p* < 0.05). The finding of an STZ-caused rise in [Ca^2+^]_i_ amount in the DRGs following TRPV4 stimulation in the patch-clamp results was further confirmed by the Fluo-3/AM data. The suppression of TRPV4 in DRGs by RESV treatments led to a decline in [Ca^2+^]_i_ concentration.

### The Injections of RESV Reduced the Increase in Oxidative Stress and Mitochondrial Dysfunction Caused by STZ

**Fig. 5:**
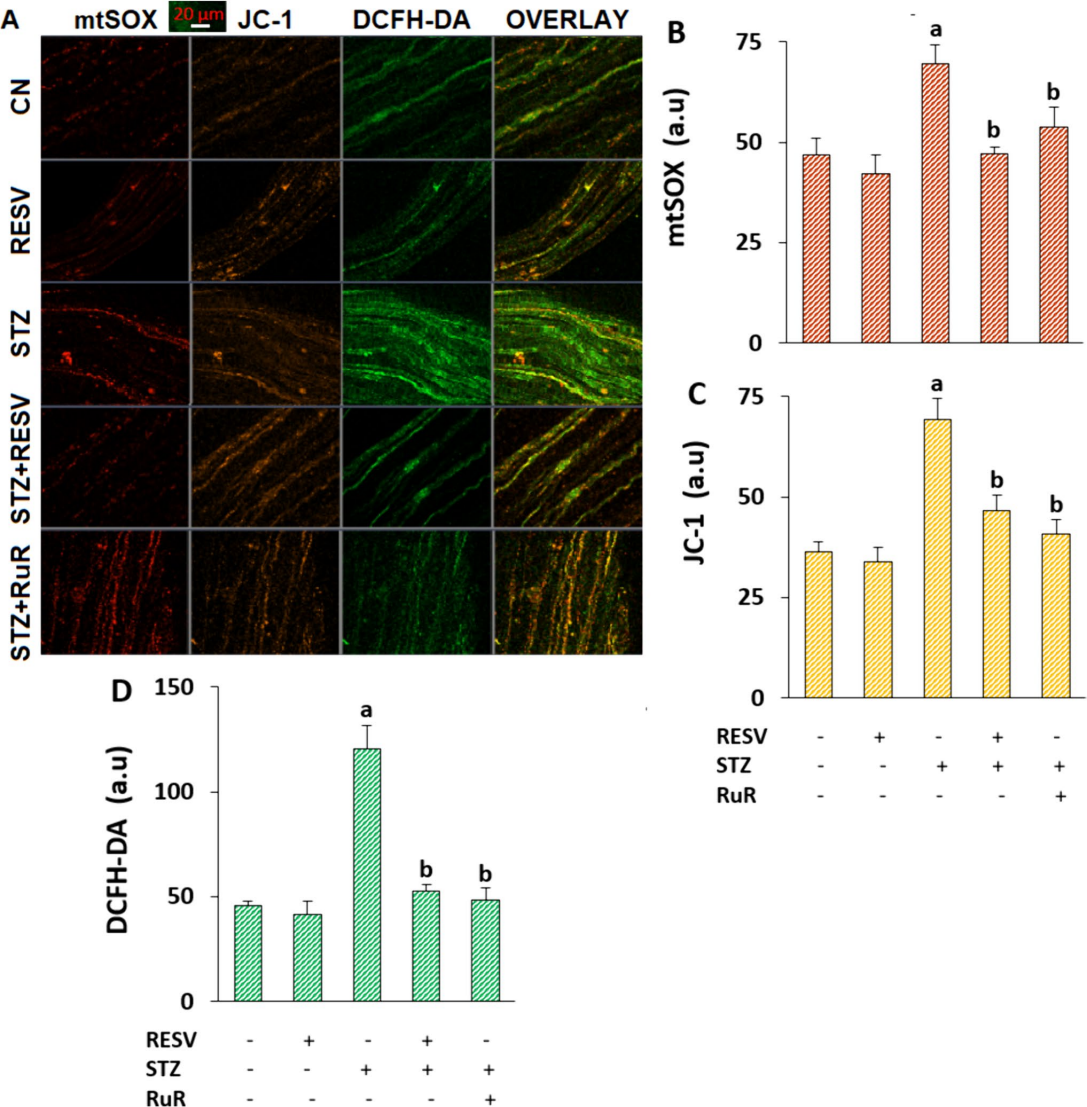
The injections of RESV (25 mg/kg for 3 weeks) reduced the STZ-induced elevation of mtSOX, mtMD, and iROS levels in the DRGs (*N* = 8, each looking at mean ± SD and 9–13 DRGs). **A** Using a 20 × 0.8 objective attached to the CSLFM, colored pictures of the stains of mtSOX (red), JC-1 (orange), DCFH-DA (green), and overlay were captured. The mtSOX (**B**), JC-1 (**C**), and DCFH-DA (**D**) mean fluorescence intensity variations expressed in arbitrary units (a.u.) (^a^*p* < 0.05 vs CN and RESV; ^b^*p* < 0.05 vs STZ)

The stimulation of TRPs, including TRPV4, leads to a decline in an opening of the mitochondrial permeability transition pore and the potential of the mitochondrial membrane, which raises mtMD in the DRGs [10, 16]. The DRGs produce more mtSOX and iROS as a result of the elevated mtMD, followed by the TRP channel-caused rise in the high Ca^2+^ entry [4, 6]. However, studies on how TRPV4-mediated Ca^2+^ influx affects mtSOX, mtMD, and iROS in the DRGs are still pending. We predicted a rise in the levels of mtSOX, mtMD, and iROS in the DRGs following the detection of upregulation in [Ca^2+^]_i_ in the DRGs of the STZ group. Images and fluorescence intensity of mtSOX (Fig. 5A and B), mtMD (Fig. 5A and C), and DCFH-DA (Fig. 5A and D) demonstrated that DRGs in the STZ group had higher levels of mtSOX, mtMD, and iROS than those in the CN or RESV groups (*p* < 0.05). However, the RuR and RESV treatments decreased the quantities in the DRGs of the STZ + RESV and STZ + RuR groups (*p* < 0.05).

### The STZ-Mediated Indicators of Apoptosis and DRG Death Were Decreased by the Injections of RESV in the DRGs

The overload increase of [Ca^2+^]_i_ amount has been linked to the control of DRG death and apoptosis [6, 9, 41]. Moreover, it has been suggested that elevated Ca^2+^ influx in the cerebral cortex of mice without diabetic mellitus has apoptotic and caspase stimulator effects, and they were modulated by the treatment of RESV [42]. In the DRGs of diabetic mice, we evaluated the levels of caspases (caspases -3, -8, and -9) and apoptosis, cell viability, and cell death (PI positive cell number) to find out whether RESV may protect the living neurons from STZ-mediated death and apoptosis.

**Fig. 6:**
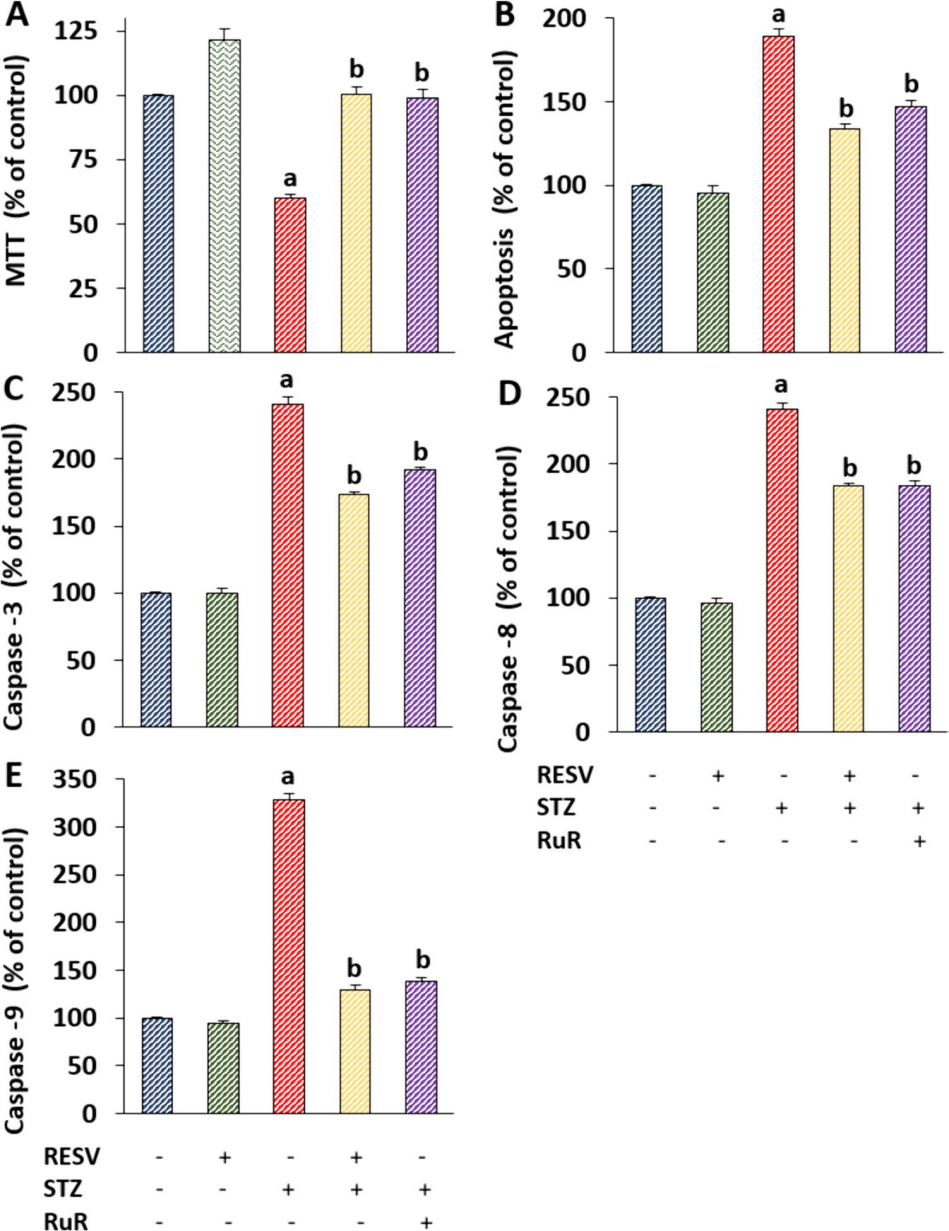
The 3-week injection of RESV (25 mg/kg) prevented the rise in apoptosis and caspases triggered by STZ (*N* = 8 and mean ± SD). Using an MTT test and a commercially available kit (APOPercantage), the plate reader (Infinite PRO 200) was used to assess the amount of cell viability (**A**) and apoptosis (**B**) in the DRGs. The caspase substrates were utilized in the plate reader (Infinite PRO 200) to determine the amounts of caspases-3 (**C**), -8 (**D**), and -9 (**E**) (^a^*p* < 0.05 vs CN and RESV; ^b^*p* < 0.05 vs STZ)

In the DRGs, STZ had a stimulator effect on the levels of apoptosis, cell viability, and caspases -3, -8, and -9. When comparing the STZ to the CN and RESV, the percentages of neuron viability were considerably (*p* < 0.05), lower (Fig. 6A). However, the addition of RuR and RESV also resulted in a substantial increase in neuron viability in the STZ + RESV and STZ + RuR groups (*p* < 0.05). The STZ group showed higher levels of apoptosis (Fig. 6B), caspase-3 (Fig. 6C), caspase-8 (Fig. 6D), and caspase-9 (Fig. 6E) in comparison to the CN and RESV. The values of STZ alone were greater (*p* < 0.05) than those of STZ + RESV and STZ + RuR.

**Fig. 7:**
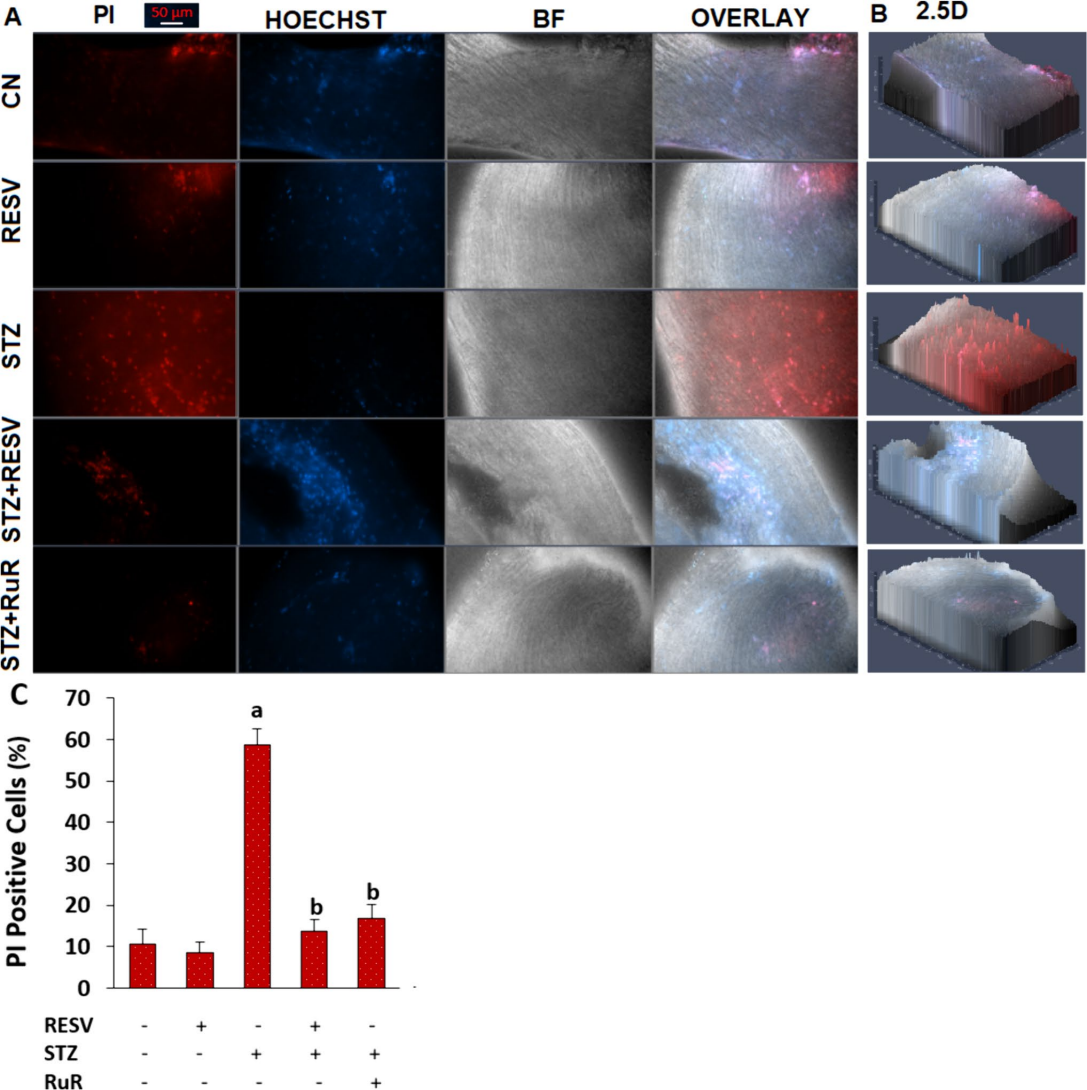
The 3-week injection of RESV (25 mg/kg) attenuated the STZ-caused increase of the death DRG percentage (*N* = 8 with each examining 9–13 DRGs and mean ± SD). **A** The images of dead (PI)/live (Hoechst 33,342) and bright field (BF) whole DRGs. **B** The 2.5D images of whole DRGs. The DRGs in the STZ + RuR group were incubated with RuR (1 µM for 1 h) after obtaining the DRGs from the STZ groups. The scale bar was kept at 50 µm. The images were captured in the CCD Axiocam 702 camera, and the data of JC-1 in the captured orange images were presented as a.u. **C** The mean percentages of PI-positive cells (^a^*p* < 0.05 vs CN and RESV; ^b^*p* < 0.05 vs STZ)

Diabetes induction enhanced the DRG death in the STZ group relative to the CN and RESV groups (*p* < 0.05), as seen by the PI-positive cell percentage in the BF (Fig. 7A), red/blue (PI/Hoechst), and 2.5D images (Fig. 7B). However, in the STZ + RESV and STZ + RuR groups, the treatments of RuR and RESV decreased the DRG death action of STZ (*p* < 0.05) (Fig. 7C).

### The Injection of RESV Influenced the STZ-Mediated Changes in Intracellular Free Zinc Ion ([Zn^2+^]_i_) Amounts in the DRGs

**Fig. 8:**
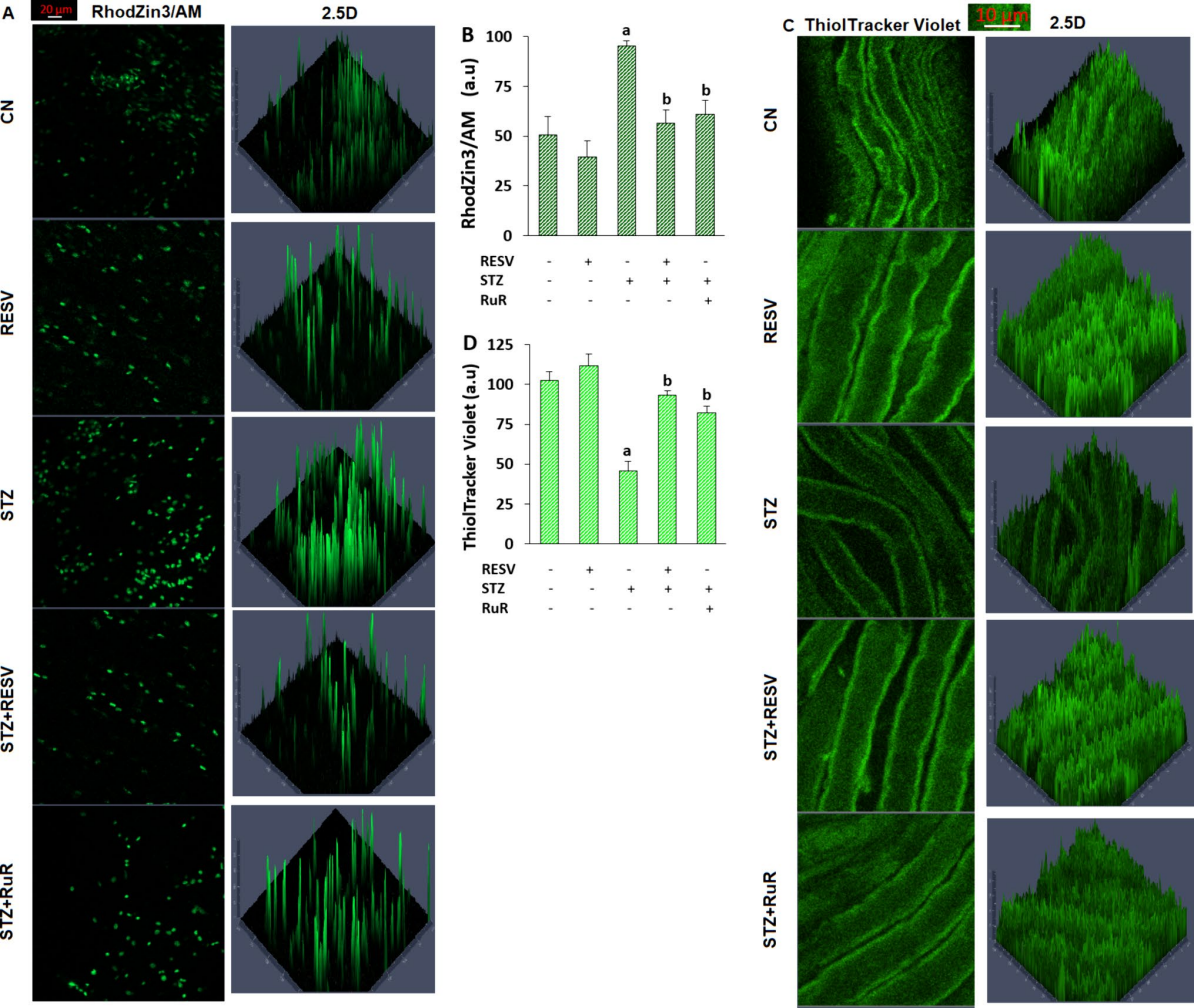
The injections of RESV (25 mg/kg for 3 weeks) reduced the STZ-induced rise in [Zn^2+^]_i_ and iGSH amounts in the DRGs (*N* = 8, each looking at mean ± SD and 9–13 DRGs). For the purpose of determining the quantities of [Zn^2+^]_i_ and iGSH, the DRGs were stained with 1 µM RhodZin3/AM (**A**) and ThiolTracker Violet (**C**). The green images were taken in the CLSFM coupled with the Plan-Apochromat 20 × /0.8 objective. **B** The RhodZin3/AM mean a.u. results. **D** The ThiolTracker Violet mean a.u. results (^a^*p* < 0.05 vs CN and RESV; ^b^*p* < 0.05 vs STZ)

Although the amounts of iGSH (Fig. 8C and D) (*p* < 0.05) were lower in the STZ group than in the CN or RESV groups, the amounts of [Zn^2+^]_i_ (Fig. 8A and B) were greater in the STZ group. In the DRGs of STZ + RESV and STZ + RuR, on the other hand, the administration of RESV caused modulator action on the STZ-induced elevations of [Zn^2+^]_i_ but decreased iGSH amounts (*p* < 0.05). On the other hand, while iGSH amounts were higher in the STZ + RESV compared to the STZ groups (*p* < 0.05), [Zn^2+^]_i_, amounts were lower in the STZ + RESV relative to the STZ groups.

### The Injections of RESV Decreased the STZ-induced Increase of LPx by Upregulating the Concentrations of rGSH, GSH-Px, Vitamin A, β-Carotene, and Vitamin E

The DRG samples were used for the other tests, so there were not enough for the remaining LPx and antioxidant assays. As a result, in the current investigation, the blood, kidney, liver, and cerebral cortex were used for the LPx and antioxidant studies rather than DRGs. The LPx content of the cerebral cortex, erythrocyte, liver, and kidney was higher in the STZ group than in the CN and RESV, while their concentrations decreased in the STZ + RESV (*p* < 0.05) (Table 1). The STZ group had lower levels of rGSH, GSH-Px, vitamin A, β-carotene, and vitamin E in the brain, erythrocytes, liver, kidney, and plasma (*p* < 0.05) compared to the CN and RESV without STZ groups (Table 1). Conversely, the administration of RESV reduced their quantities in the STZ + RESV (*p* < 0.05).

**Table 1:** The beneficial effects of resveratrol (RESV) on various antioxidant vitamin levels, lipid peroxidation (LPx), reduced glutathione (rGSH), and the activity of glutathione peroxidase (GSH-Px) were studied in the brain, erythrocyte, liver, kidney, and plasma of STZ-induced diabetic mice (mean ± SD)

	Values	Control (*n* = 8)	RESV (*n* = 8)	STZ (*n* = 8)	STZ + RESV (*n* = 8)
LPx*	Brain	21.62 ± 1.13	19.89 ± 0.62	26.56 ± 2.30^a^	20.69 ± 1.77^b^
	Erythrocyte	16.60 ± 1.34	15.28 ± 0.15	21.07 ± 1.84^a^	16.51 ± 1.29^b^
	Kidney	15.18 ± 1.42	13.00 ± 2.13	19.62 ± 1.54^a^	16.31 ± 1.75^b^
	Liver	19.33 ± 1.95	18.73 ± 1.54	23.47 ± 1.16^a^	20.10 ± 1.81^b^
rGSH*	Brain	15.60 ± 1.30	17.00 ± 1.21	13.80 ± 0.60^a^	15.80 ± 0.30^b^
	Erythrocyte	18.30 ± 0.33	19.10 ± 1.88	16.30 ± 0.16^a^	18.40 ± 1.50^b^
	Kidney	12.40 ± 1.19	13.00 ± 0.87	9.30 ± 0.55^a^	12.30 ± 1.34^b^
	Liver	13.00 ± 1.13	14.70 ± 0.99	11.40 ± 0.47^a^	13.10 ± 0.35^b^
GSH-Px^≠^	Brain	29.40 ± 1.17	30.50 ± 2.31	22.70 ± 1.15^a^	27.30 ± 1.85^b^
	Erythrocyte	25.60 ± 2.20	29.00 ± 1.37	18.40 ± 1.48^a^	24.00 ± 1.96^b^
	Kidney	21.50 ± 1.71	22.50 ± 1.28	17.90 ± 1.94^a^	21.10 ± 2.33^b^
	Liver	24.80 ± 2.38	25.30 ± 0.50	19.30 ± 1.11^a^	25.60 ± 1.62^b^
α-Tocop^●^	Brain	20.20 ± 1.38	21.60 ± 1.49	15.60 ± 1.16^a^	18.20 ± 1.46^b^
	Kidney	12.20 ± 1.45	13.40 ± 0.80	9.18 ± 1.06^a^	12.70 ± 1.49^b^
	Liver	25.10 ± 1.07	26.00 ± 0.66	21.10 ± 1.49^a^	25.20 ± 1.07^b^
	Plasma^Χ^	22.20 ± 1.10	25.60 ± 1.76	18.40 ± 1.17^a^	21.70 ± 1.45^b^
Retinol^●^	Brain	3.68 ± 0.45	4.08 ± 0.59	3.28 ± 0.44^a^	3.77 ± 0.39^b^
	Kidney	3.56 ± 0.57	3.93 ± 0.62	3.31 ± 0.30^a^	3.68 ± 0.49^b^
	Liver	25.10 ± 1.07	26.00 ± 0.66	21.10 ± 1.49^a^	25.20 ± 1.07^b^
	Plasma^Χ^	22.20 ± 1.10	25.60 ± 1.76	18.40 ± 1.17^a^	21.70 ± 1.45^b^
β-Car^●^	Brain	1.54 ± 0.26	1.56 ± 0.25	1.26 ± 0.09^a^	1.54 ± 0.16^b^
	Kidney	1.49 ± 0.12	1.70 ± 0.25	1.29 ± 0.10^a^	1.50 ± 0.14^b^
	Liver	2.56 ± 0.47	3.43 ± 0.49	2.17 ± 0.13^a^	2.67 ± 0.14^b^

^a^*p* < 0.05 vs control and RESV; ^b^*p* < 0.05 vs STZ)

^*^µM/g protein

^≠^IU/g protein

^●^µM/g tissue

^Χ^µM/l

## Discussion

Through upregulation of blood glucose, DRG neuron mtMD, LPx, iROS, mtSOX, apoptosis, caspases, and TRPV4-caused [Ca^2+^]_i_ rise in the DRGs of mice, we have demonstrated in the present study that the STZ treatment significantly stimulated the development of mechanical and thermal DPN, although they were decreased by the treatment with the RESV and selective TRPV4 channel antagonist (RuR). The treatments for RESV and RuR exacerbated the STZ-mediated reductions of neuron viability, iGSH, rGSH, GSH-Px, α-carotene, and vitamins A and E. In diabetic mice, the anti-DPN impact of RESV appears to be caused by the inhibition of TRPV4 channels.

Complications associated with DPN have been extensively investigated with the STZ-mediated type 1 diabetes mellitus model [9, 10]. Furthermore, thermal hyperalgesia caused by diabetes can be investigated in experimental animals [11]. Increases in Ca^2+^ influx and STZ-mediated oxidative stress cause DPN and mechanical hyperalgesia [4–7]. STZ-mediated oxidative stress stimulates the TRPV4 channel; however, antioxidant therapies reduce the activation of TRPV4-mediated Ca^2+^ influx by inhibiting oxidative stress [10, 18]. Through the regulation of TRP channels like TRPM2 and TRPM7 in neuronal cells [25] and prostate cancer cells [41], the RESV therapy altered Ca^2+^ influx. It has also been documented that RESV protects against STZ-induced elevations in blood glucose, temperature, and mechanical DPN in rats [43]. However, the mechanism by which RESV protects against DPN in mice via TRPV4 channel regulation remains unclear. In the current study, mice who received an injection of STZ had greater blood glucose levels and mechanical and thermal DPN for 3 weeks, but they also significantly reduced their weight increase in comparison to control mice. Nonetheless, an injection of RESV regulated the alterations by inhibiting TRPV4.

A large number of TRP channels, such as TRPV4, lack specific antagonists. RuR, HC-067047, and carvacrol are the most widely used TRPV4 antagonists [14, 15, 20, 21]. RuR was chosen as a TRPV4 antagonist in the current study for the following reasons: The majority of research on TRPV4 antagonists focuses on RuR-induced TRPV4 inhibition, as seen in numerous publications. Furthermore, it was noted that there is an allosteric connection between conformational changes at the lower gate and RuR binding at the selective filter in TRPV4 [44]. In addition to TRPV4, RuR inhibits a variety of cation channels, such as several TRP channels (i.e., TRPA1, TRPM8, and TRPV1), calcium homeostasis modulator channels, ryanodine receptors, and Piezo channels [15, 20, 21, 44]. This is a disadvantage of RuR for the TRPV4 studies.

In the mice cultured primary liver cells and SH-SY5Y cells, an excessive amount of Ca^2+^ influx via the stimulation of TRPV4 elevated the amounts of oxidants (LPx, iROS, and mtSOX) [21, 45]. However, through the blockage of the TRPM2 in the neurons of laboratory animals with diabetes mellitus, antioxidant therapies, such as the RESV, decreased the amount of [Ca^2+^]_i_ and oxidants [4, 25, 46]. It remains, however, not known whether oxidants and the [Ca^2+^]_i_ amount caused by TRPV4 activation interact in the DRGs of mice suffering from diabetes mellitus. The current study evaluated how STZ-injection-induced [Ca^2+^]_i_ amount in DRGs was enhanced by TRPV4 stimulation. The STZ group exhibited higher levels of LPx, iROS, and mtSOX in the DRGs. When mice were co-treated with RESV, the generations of LPx, iROS, mtSOX, and [Ca^2+^]_i_ amounts declined with the inhibition of TRPV4. It appears that the injections of RESV lowered the STZ-induced increase in oxidant levels and [Ca^2+^]_i_ amount in the DRGs. According to the findings, in the DRGs of mice and rats with diabetes mellitus, injections of antioxidants such as selenium, melatonin, and Noopept reduced the oxidant, TRPM2, TRPM7, and TRPV1 stimulator effects of STZ [4, 6, 8]. In the sciatic nerve and DRGs of mice, the injection of antioxidant (phenyl-α-tert-butyl nitrone) and TRPV4 inhibitors (HC-030031 and HC-067047) decreased the oxidants (H_2_O_2_ and 4-hydroxynonenal) and DPN caused by thalidomide and chemotherapy agents [14].

One important regulator of the Krebs cycle and oxidative phosphorylation in the mitochondria is the amount of [Ca^2+^]_i_ [47]. Ca^2+^ and Zn^2+^, as cofactors of antioxidant enzymes and proteins, induced mtSOX scavenger action [47]. However, high amounts of free [Ca^2+^]_i_ and [Zn^2+^]_i_ in the mitochondria stimulate the production of mtSOX and activate pro-apoptotic signals through caspase-3, caspase-8, and caspase-9 in the brain and neurons, including DRGs [4, [4, 6, 8, 47, 48]. Increases in mtMD occur when there is an opening in the mitochondrial permeability transition pore and an increase in mitochondrial swelling due to huge accumulations of [Ca^2+^]_i_ and [Zn^2+^]_i_ [47, 48]. The brain and neurons undergo an increase in [Ca^2+^]_i_ due to the activation of TRPV4, an oxidative stress–dependent TRP channel [10]. Supporting evidence for this hypothesis are the present findings, which demonstrate that elevated [Ca^2+^]_i_ and [Zn^2+^]_i_ amounts resulting from TRPV4 activations in diabetic conditions eventually damage the mitochondrial membranes. This may be a factor in the rise in mitochondrial dysfunction (mtMD and mtSOX) and LPx observed in DRG neurons. By upregulating the PI-positive cell numbers, caspase-3, 8, and 9 activities but downregulating those of iGSH, rGSH, and GSH-Px, they in turn induced DRG death and apoptosis [6, 9]. Taken together, these findings show the importance of TRPV4-stimulated Ca^2+^ entry as a common mediator in situations involving DRG death or DRG survival.

For the synthesis of members of the thiol redox system, including iGSH, rGSH, and GSH-Px, cysteine is a necessary source [49]. Redox alteration of the free thiol groups is the most prevalent method by which ROS directly activates the TRP channels, including TRPV4 [50, 51]. Consequently, the activation of TRPV4 is affected by the change in cysteine residues in cells. Therefore, the TRPV4 channel is stimulated by downregulation of iGSH, rGSH, and GSH-Px [21, 52], whereas the TRPV4 channel is inhibited by overexpression of iGSH, rGSH, and GSH-Px via antioxidant therapy [21, 52]. On the subject, it was reported that ROS scavengers (Trolox or ARL-17477) significantly reduced GSK-induced neuronal mortality and apoptosis through an elevated level of nitric oxide but downregulation of GSH-Px and catalase in the hippocampus CA1 region of mice [52]. In mouse sensory neurons, paclitaxel-induced elevations in mechanical allodynia, mtSOX levels, and TRPV4 activity were further amplified by a reduction in GSH [53]. In both humans and laboratory animals, TRPV4 stimulation leads to complications associated with diabetes [54]. Antioxidant therapies have been shown to modulate TRPV4 [10], yet there is an inconsistent report as well [55]. Hypoxia-induced increases in neuronal cell death, apoptosis, caspases, and mitochondrial oxidative stress were decreased in the SH-SY5Y neuronal cells by the treatment of TRPV4 agonist (GSK) [56]. Through the regulation of the TRPM2 channel in the SH-SY5Y neuronal cells, the incubation of RESV reduced hypoxia-induced neuronal cell death, apoptosis, caspases, and mitochondrial oxidative stress [25]. Similar anti-apoptotic and antioxidant effects of RESV were noted on diabetes mellitus-mediated oxidative retinopathy in mice through TRPM2 channel regulation [46]. The TRPV4 blocker effect of RESV was studied in order to further confirm the involvement of Ca^2+^ signaling in STZ-mediated apoptosis, mitochondrial dysfunction, and antioxidant deficiencies. Based on our findings, STZ-induced apoptosis, dysfunction of the mitochondria, and a drop in levels of antioxidants in the DRGs of mice were all prevented by RESV. Specifically, the injections of RESV restored STZ-mediated elevations of TRPV4 stimulation; caspase-3, caspase-8, and caspase-9; iGSH, rGSH, and GSH-Px, as well as vitamin A, α-carotene, and vitamin E levels. The current data provide more evidence that the overexpression of caspases, but the reduction of antioxidants during STZ-induced DRG death and apoptosis is primarily due to TRPV4 activation-mediated increases in [Ca^2+^]_i_ concentration, mtMD, and mtSOX. On the other hand, reduction of caspases but overexpression of antioxidants was the mechanism by which the RESV injections caused DRG protection.

Because of their high oxygen consumption rate and high polyunsaturated fatty acid composition but low antioxidant quantity, the brain and erythrocytes are particularly susceptible to oxidative damage [57]. In the experimental animals, STZ-mediated oxidative stress also primarily damages the liver and kidney [4, 35]. However, this effect was minimized by the treatment of rats with RESV, which increased the levels of vitamin E, rGSH, and GSH-Px in the liver [58]. Vitamin E and β-carotene, which are fat-soluble antioxidant vitamins, have the ability to scavenge different radicals, including hydroxyl and singlet oxygen radicals [56]. Through the downregulation of LPx in the plates of patients with type II diabetes, the addition of RESV increased GSH and cell viability [59]. The brain, erythrocyte, kidney, liver, and plasma of diabetic experimental individuals and animals were found to have decreased concentrations of both enzymatic (GSH-Px) and non-enzymatic (iGSH, rGSH, vitamin A, vitamin E, and β-carotene) antioxidants [4, 35, 59]. In the present investigation, the administration of RESV treatment increased the STZ-induced reductions of both enzymatic and non-enzymatic antioxidants in the blood and tissue samples. The blood and tissue samples exhibited greater amounts of both enzymatic and non-enzymatic antioxidants as a consequence of the mtROS scavenger activity of RESV.

When combined, these data demonstrate that using STZ to induce diabetes mellitus results in the DPN by triggering the oxidative and apoptotic dysfunctions of the mitochondria and lowering enzymatic and non-enzymatic antioxidants and cell viability. The stimulation of TRPV4 resulted in an increase in Ca^2+^ signaling and mtROS pathways, which in turn generated DPN. Conversely, by inhibiting TRPV4 in the DRGs, the RESV injections prevented the DPN, oxidant, and apoptotic harmful effects of STZ. Our data support the hypothesis that the TRPV4 channel could be an effective target to treat PDN, and the injection of RESV has an effective action on the DPN by inhibiting the oxidant, apoptotic, and TRPV4 stimulator actions of STZ in the DRGs. As a result, using the RESV to modulate TRPV4 may be an effective new method for treating DM-caused DPN, oxidative DRG damage, and apoptosis. In future studies, detailed evaluations of the influence of STZ and RESV on neuropathic pain before investigating their effects on Ca^2+^-mediated mitochondrial dysfunction, ROS production, and DRG neuron viability will be evaluated separately.

## Data Availability

The treatments and animal tissue samples in the current study were started using MAKU. Dr. M. Nazıroğlu can provide the results of the analyses upon request. BSN Health, Analyses, Innovation, Consultancy, Organization, Agriculture, and Industry Ltd. (Göller Bölgesi Teknokenti, Isparta, Türkiye) was the location of the analyses. All the visuals in the manuscript, including the graphical abstract, were induced by the correspondence author (MN).

## Abbreviations

[Ca^2+^]_i_: Cytosolic free calcium ion
CLSFM: Confocal laser scanning fluorescence microscope
DPN: Diabetic peripheral neuropathy
DRG: Dorsal root ganglion
GSH-Px: Glutathione peroxidase
GSK: GSK1016790A
iGSH: Intracellular glutathione
iROS: Intracellular reactive free oxygen radicals
LPx: Lipid peroxidation
mtMD: Membrane depolarization of mitochondria
mtSOX: Reactive free oxygen radicals of mitochondria
RESV: Resveratrol
rGSH: Reduced glutathione
RuR: Ruthenium red
STZ: Streptozotocin
TRP: Transient receptor potential
TRPV4: Transient receptor potential vanilloid 4

## Declarations

### Accessibility of Information and Resources

The treatments and animal tissue samples in the current study were started using MAKU. Dr. M. Nazıroğlu can provide the results of the analyses upon request. BSN Health, Analyses, Innovation, Consultancy, Organization, Agriculture, and Industry Ltd. (Göller Bölgesi Teknokenti, Isparta, Türkiye) was the location of the analyses. All the visuals in the manuscript, including the graphical abstract, were prepeared by the communication author (MN).
